# Applying P-Technique Factor Analysis to Explore Person-Specific Models of Readiness-to-Exercise

**DOI:** 10.3389/fspor.2021.685813

**Published:** 2021-06-25

**Authors:** Kelley Strohacker, Richard Keegan, Cory T. Beaumont, Rebecca A. Zakrajsek

**Affiliations:** ^1^Kinesiology, Recreation, and Sport Studies, The University of Tennessee, Knoxville, Knoxville, TN, United States; ^2^Research Institute for Sport and Exercise Science, Faculty of Health, University of Canberra, Bruce, ACT, Australia

**Keywords:** idiographic analysis, ecological momentary assessment, interpersonal signatures, subjective assessment, individualization

## Abstract

Recent research in exercise prescription and periodization has emphasized the importance of subjective experience, both in medium- and long-term monitoring, but also in the acute experience. Emerging evidence also highlights an important role of subjective readiness (pre-exercise mental and physical states) in determining how exercise is experienced, and in acutely modifying the prescribed exercise intensity. The concept of “readiness-to-exercise” shows promise in enabling and informing this acute decision-making to optimize the experiences and outcomes of exercise. While subjective experiences can be effectively assessed using psychometric scales and instruments, these are often developed and deployed using cross-sectional samples, with resulting structures that reflect a normative pattern (nomothetic). These patterns may fail to reflect individual differences in sensitivity, experience and saliency (idiographic). We conducted this research with the primary aim of comparing the nomothetical and idiographic approaches to modeling the relatively novel concept of readiness-to-exercise. Study 1 (nomothetic) therefore analyzed data collected from 572 participants who completed a one-time survey using R-technique factor analysis. Results indicated a four-factor structure that explained 60% of the variance: “health and fitness;” “fatigue;” “vitality” and “physical discomfort.” Study 2 (idiographic) included a sample of 29 participants who completed the scale multiple times, between 42 and 56 times: permitting intra-individual analysis using separate P-technique factor analyses. Our analyses suggested that many individuals displayed personal signature, or “profiles” of readiness-to-exercise that differed in structure from the nomothetic form: only two participants' personal signatures contained four structures as modeled in Study 1, whereas the majority demonstrated either two or three factors. These findings raise important questions about how experiential data should be collected and modeled, for use in research (conceptual development and measurement) and applied practice (prescribing, monitoring)—as well as in more applied research (implementation, effectiveness).

## Introduction

Exercise, which refers to planned, structured, and repetitive activity to improve fitness, is commonly promoted as a means of increasing total physical activity levels and eliciting numerous health benefits, such as reduced risk of cardiometabolic disease and certain cancers (Febbraio, 2017). Yet, like many complex health behaviors, poor long-term adherence to exercise represents a persistent problem (Martin et al., 2005; Middleton et al., 2013). To facilitate regular behavioral engagement, service providers should ensure that the structure of the programming components (mode, frequency, intensity, duration, volume, progression; i.e., the “prescription”) are optimally designed (i.e., maximizing physiological adaptation and minimizing risk) and clearly communicated (American College of Sports Medicine, 2017). Additionally, the prescription components should be matched to each individual's health status, exercise responses, and stated goals (Garber et al., 2011) to provide a basic foundation for physiological adaptation and behavioral maintenance (King and Senn, 1996; Zubin Maslov et al., 2018; Weatherwax et al., 2019). That is, individuals are unlikely to adhere to a program that yields minimal salient benefits, or to a program they find too challenging to perform as directed. To further support long-term maintenance, Ekkekakis et al. (2011) proposed that prescription components, particularly intensity, should also be designed to minimize negative affective responses (i.e., displeasure) during exercise, as a such responses predict lower future activity levels (Brand and Ekkekakis, 2018).

Individuals will also continually adapt to training stimuli, meaning that exercise prescription is best approached as a dynamic process where programmatic modifications must be responsive to emerging information (Kraemer and Ratamess, 2004; Sasso et al., 2015). While the standard attributes upon which we develop exercise prescriptions (health status, fitness level, and long-term goals) may be less likely to undergo rapid, unpredictable fluctuations, they are also not inherently stable and require assessment and subsequent modification several times per year. Conversely, modifications to optimize exercise *experiences* should occur more acutely. Recent evidence has demonstrated that in-task affect is not solely influenced by intensity, but also by pre-exercise affective states (e.g., moods, emotions, energy/tiredness), physical condition (e.g., residual soreness, illness, pain) and cognitions (e.g., perceived self-efficacy, anticipated affect; Rose and Parfitt, 2007, 2010; Vazou-Ekkekakis and Ekkekakis, 2009; Sala et al., 2016; Zenko et al., 2016; Strohacker et al., 2017; Beaumont et al., 2021). These and other physical, psychological, cognitive, and contextual correlates of exercise behavior can fluctuate relatively frequently over time (Dunton and Atienza, 2009; Dunton, 2017), potentially due to change in other behaviors (sleeping, eating) or events (bad news). Citing the failure of current health-based models to promote sufficient physical activity Barreto (2013) proposed that exercise should be promoted “with the flexibility of being adaptable to a person's circumstances” (p. 390). Similarly, noting that poor behavioral outcomes following theory-driven interventions are likely due to “one-size-fits-all” approaches, Conroy et al. (2020) proposed that “the science of physical activity promotion will advance most rapidly if person-specific psychological, contextual, and behavioral dynamics can be leveraged to adapt or ‘tune’ interventions to the specific requirements of each individual” (pp. 171).

“Autoregulation” (i.e., the purposeful and frequent adjustment of programming that corresponds to measurable changes in an individual's response to training- and non-training-related stressors) is a recognized and continually developing concept within competitive sport contexts (Greig et al., 2020). The routine monitoring of stressors is purported to guide training in a way that maximizes performance outcomes, reduces risk of negative acute experiences (e.g., overtraining, injury, incompletion, and psychological distress), and minimizes training response variance (Kraemer and Fleck, 2007; Borresen and Lambert, 2009; Mann et al., 2014; Thorpe et al., 2017). Flexible Nonlinear Periodization (FNLP) is an autoregulation strategy where training workloads, which are goal-specific and range from low- to high-demand, are chosen each day based on each individual's “readiness-to-train” (pre-exercise mental and physical states; Kraemer and Fleck, 2007). While Greig et al. (2020) suggest that autoregulation strategies may be beneficial in health-promotion settings, the authors note that the implementation of these exercise frameworks is limited in the health domain.

A particularly glaring gap relates to the operationalization of “readiness.” In their foundational text, Kraemer and Fleck (2007) provided a six-factor checklist (coach-trainee interactions; injury status; hydration; mental and physical fatigue; vertical jump power; initial workload performance), whereas Mann et al. (2014) suggested that training status, sleep, stress, and habitual physical activity would indicate readiness-to-train. While these indices make intuitive sense, neither source presented empirical evidence to support their use in training scenarios. Within the existing FNLP literature, conceptualizations of readiness-to-train have varied, such that participants had been instructed to consider their energy level alone (McNamara and Stearne, 2010), reflect upon a combination of factors (e.g., mood, preference, energy, diet, stress level; McNamara and Stearne, 2013), or were not explicitly provided with indices to consider (Colquhoun et al., 2017) before choosing their training bout each day. Rationales were not provided to support these approaches and no data were presented to explore whether or not the suggested indices of readiness-to-train were actually utilized or, in the case of the latter study, what factors ultimately influenced participants' day-to-day decisions. These omissions are problematic because, on one hand, identifying a single marker of readiness-to-exercise assumes that it holds equal importance to all participants. On the other hand, providing multiple indices of readiness-to-train (or none at all) removes the researchers' burden to identify each individual's most salient construct(s). But, this approach assumes that: (1) individuals are cognizant of what factors most impact their exercise performance; and (2) that individuals will act in good faith, in accordance with their level of readiness. Without evidence of these assumptions, we might alternatively propose that individuals left to their own volition may choose workloads that are actually incongruent with their current state of readiness, which would compromise the fidelity of the novel FNLP training paradigm. Such ambiguity regarding target indices of readiness and participants' compliance (i.e., choosing lower-demand bouts when presenting with reduced readiness) severely limits researchers' ability to empirically test behavioral, psychological, and physiological effects of FNLP as a strategy for personally-adaptive exercise programming.

In an effort to address this gap, researchers have demonstrated initial, empirical evidence for target indices underlying perceptions of readiness in non-athlete sample populations, which we will herein refer to as “readiness-to-exercise” to denote a conceptual shift toward health promotion rather than sport-specific training. An initial study, conducted using survey data from university students, presented four dimensions of readiness-to-exercise labeled “vitality” (positive mood, energy), “fatigue,” “discomfort” (illness, soreness), and “health/fitness” (Strohacker and Zakrajsek, 2016). These dimensions were similarly reflected in a subsequent study using thematic analyses, which demonstrated that adults with obesity indicated that their readiness-to-exercise is impacted by perceptions of affective valence and activation (accounting for mood/emotional states and feelings of energy/fatigue), body integrity (injury, sickness, and soreness), physical fitness, fuel (hydration and food intake), and motivation (Strohacker et al., 2019). These shared dimensions of readiness-to-exercise not only reflect the prior (though not empirically-based) conceptualizations of readiness-to-train from the strength and conditioning literature, but also demonstrate overlap with the aforementioned determinants of exercise-related affect. As such, these dimensions, which represent vitality (e.g., energetic valence, mood/emotional state), fatigue, physical discomfort, and perceptions of health and fitness, as well as their structure served as target factors for the current study. We note here that many of these indicators may be measured objectively, and yet subjective indicators have also been shown to outperform, or at least complement such objective measures (Saw et al., 2016).

However, an important limitation of this work is that these conceptualizations of readiness-to-exercise were determined by generalizing responses *across* individuals, potentially obscuring or ignoring the scope for individual differences. Researchers often deploy the “top-down” approach (i.e., inferring idiographic properties based on nomothetic analyses) for data interpretation and application. Contrasting the common assumption in research that idiographic properties can be inferred based on nomothetic analysis, Molenaar (2004) examined psychological variables using mathematical modeling procedures for both approaches. This work demonstrated that structural features of data derived by analyzing *inter*individual variation cannot be generalized to data derived by analyzing *intra*individual variation. Thus, at minimum, it is important to examine both between-person and within-person structures regarding readiness-to-exercise factors prior to developing and implementing FNLP-based exercise programming.

Examining the structural features of multivariate data is often accomplished through factor analysis, which assumes that patterns of covariation among measured variables can be explained using fewer latent constructs. The most widely applied approach, R-technique, is used to examine factor structure at a population level by modeling single-observation data collected from a large number of individuals (Cattell, 1952). Results provide insight into the number of factors, total variance in the data explained by each factor, correlations between factors, pattern of measured items loading on each factor, and magnitude and direction of each item's factor loading. These same procedures can also be applied to time series data to examine the structure of multivariate data *within an* individual, a process known as P-technique factor analysis (Cattell et al., 1947; Cattell, 1963). Molenaar and Nesselroade (2009) demonstrated that the ability of this approach to recover underlying factors is comparable to that of dynamic factor modeling regarding accuracy and robustness. Researchers applying P-technique factor analyses to psychometric data (generally relating to personality research) have also demonstrated that individual factor structures can be relatively diverse compared to results uncovered using R-technique (Lebo and Nesselroade, 1978; Borkenau and Ostendorf, 1998; Molenaar, 2004; Fournier et al., 2008, 2009; Wright et al., 2016), resulting in unique structural features, or, as coined by Fournier et al. (2009), “interpersonal signatures.” Wright et al. (2016) surmised that the ability to evaluate such personal “signatures” may help practitioners and clinicians better individualize treatment plans. To date, the degree of diversity present within personalized factor structures pertaining to readiness-to-exercise is a novel research question that has yet to be examined.

The purpose of the current study was to examine nomothetic and idiographic structural features of factors underlying readiness-to-exercise. Two studies were conducted using two existing databases to answer the following research questions: “what is the structure of readiness-to-exercise factors measured in a pre-exercise context?” and “what level of heterogeneity is observed among interpersonal structures of readiness-to-exercise factors measured over time?” Both databases included the same 12 items chosen to represent four dimensions of readiness-to-exercise as previously operationalized by Strohacker and Zakrajsek (2016).

## Materials and Methods

All methods described herein were approved by the University Institutional Review Board (IRB): Study 1 IRB #14-01841, Study 2 IRB #16-03048-XP.

### Study 1—R-Technique Factor Analysis to Determine a Reference Structure for Readiness-to-Exercise

#### Participants and Sample Size Considerations

We sought a convenience sample of participants (faculty, staff, and students at a large university in the Southeast region of the United States) between January and April 2015 at the university recreation center. We focused our sampling on individuals who were at least 18 years of age and were at the recreation center to exercise (as opposed to meeting friends, purchasing food and drinks, for work, etc.). Sampling is considered to be adequate if the Kaiser-Meyer-Olkin (KMO) test >0.50 and Bartlett's test of sphericity reveals statistical significance (*p* < 0.05) (Field, 2005). Regarding sample size, the ratio of observations (572) to variables (12) was 47:1. This value is considered adequate for factor analysis (Myers et al., 2011).

#### Procedures

We approached individuals after entering the university recreation building, but before swiping their identification cards to access areas containing fitness equipment. Interested individuals were provided with a written study information sheet that outlined details regarding the purpose, expectations, and risks of participating in the research study. Individuals were not asked to provide any identifying information, thus assuring participant anonymity. Individuals were also informed that completion of the pen-and-paper survey served as their consent to participate. A research assistant reviewed each survey. In the event of a skipped question, the research assistant asked the respondent to address the error. However, in cases where whole sections were left unanswered, the research assistants interpreted this as withdrawn consent and discarded the survey. We also discarded surveys that indicated the participant was under the age of 18. Of the initial 602 returned surveys, there were 30 surveys discarded due to the above criteria. A total of 572 surveys were retained for statistical analyses.

#### Instrumentation

##### Indices of Readiness-to-Exercise

Using a “right now” prompt, participants were asked to rate 12 items using a seven-point Likert scale (0 = not at all, 3 = moderately, 6 = extremely). In the original scale development, these items (indicated in parentheses) were chosen to represent the four factors of readiness-to-exercise: “vitality” (energetic, happy, and lively), “fatigue” (worn out, exhausted, and drained), “discomfort” (pain, achy, and stiff), and “health/fitness” (healthy, fit, and strong) as determined by a previous study (Strohacker and Zakrajsek, 2016). The instrument was limited to three items per factor for brevity and avoid potential redundancy; items were chosen based on ease of understanding, in that items were thought to be most representative of the related factor (Burisch, 1984).

##### Habitual Exercise

Respondents were first asked to indicate how many days per week they currently exercised. Those indicating one or more days per week were further asked to indicate how many minutes per day they spend exercising, on average. These values were multiplied to calculate total minutes per week of exercise.

##### Demographics

Participants were asked to indicate their age, current position at the university (undergraduate student, graduate student, staff member, and faculty member), gender, race, and ethnicity.

#### Statistical Analyses

Statistical procedures were conducted using the Statistical Package for the Social Sciences (SPSS; IBM, Armonk, NY). Means, standard deviations, and ranges were computed for all items. Raw data were not transformed or standardized prior to performing the factor analysis. The factor analysis was conducted using the principal axis method, as a number of variables demonstrated non-normal patterns of distribution (Fabrigar et al., 1999), and oblique (promax) rotation was applied, as we expected a degree of correlation between factors (Costello and Osborne, 2005). We conducted an initial analysis retaining factors based on eigenvalues (factors with values ≥1.0 were retained) to determine suitability for conducting factor analysis using the KMO and Bartlett's test of sphericity. Using available syntax (O'Connor, 2000), we then conducted a parallel analysis of the data to statistically determine the number of factors to retain. Raw data eigenvalues were considered significant (and thus, retained as factors) if they were larger than the 95th percentile eigenvalues and larger than the mean random data eigenvalues. We then examined the structural features of the resultant model (number of factors, proportion of variance explained, between-factor correlation, and item loading).

### Study 2—P-Technique Factor Analysis to Examine Heterogeneity in Interpersonal Structures of Readiness-to-Exercise

#### Participants and Sample Size Considerations

A separate sample of participants from the same southeastern university were recruited *via* flier advertisements, listservs, and word of mouth between June and November 2016 to undergo ecological momentary assessment of exercise behavior and hypothesized correlates. Regardless of current exercise behavior, individuals were eligible to participate if they: (1) were at least 18 years old; (2) were not varsity athletes; and (3) owned a smartphone with text messaging and internet capabilities. Overall, 29 participants consented and completed all study procedures. Participants in this study provided 42–56 points of observation per person (Mean ± SD = 50 ± 4) for 12 items (mean observation-to-item ratio = 4:1). Molenaar and Nesselroade (2009) previously assessed the robustness of the P-technique using sample sizes (i.e., number of data points observed within a person) of 300 observations, 100 observations (considered a general rule of thumb), and 50 observations (a more realistic number of observations in longitudinal research studies). Similar results were found regarding factor loading pattern and strength (but with larger standard deviations) when comparing 50 observations and 300 observations relative to the true loading structure.

#### Procedures

Interested individuals were invited to an in-person session with a member of the research team. Eligible individuals who provided voluntary consent to participate underwent objective measures of height and weight to calculate body mass index (BMI) using standard procedures and then completed a baseline survey to assess demographic characteristics.

Participants were then familiarized with the primary survey designed to assess exercise behavior and correlates thereof. This survey was built and distributed using Qualtrics Research Suite (Provo, UT). In line with the accepted definition of “exercise” (Caspersen et al., 1985), participants were explicitly asked to only report activities as exercise if they were planned, structured, and performed with the purpose of maintaining and improving one or more component of physical fitness. Additionally, we explicitly asked participants to avoid reporting non-exercise activity (e.g., transportation, chores, and work). For 14 consecutive days, participants received short-code text messages to their smartphone that contained an Internet link to open and complete the survey. Text prompts were sent four times per day at 9:30 am, 1:30 pm, 5:30 pm, and 9:30 pm. All participants who completed the study received $20 in grocery store gift cards.

#### Instrumentation

##### Indices of Readiness-to-Exercise

Using a “right now” prompt, participants were asked to rate 12 items using a seven-point Likert scale (0 = not at all, 3 = moderately, 6 = extremely). The items and rating format used here were identical to those in Study 1 and based on a previous dimensionality of readiness-to-exercise (Strohacker and Zakrajsek, 2016) to address “vitality” (energetic, happy, and lively), “fatigue” (worn out, exhausted, and drained), “discomfort” (pain, achy, and stiff), and “health/fitness” (healthy, fit, and strong), These items were included in each electronic survey.

##### Exercise Behavior

In response to the prompt “in the past 4 h, did you perform any of the following exercises?” participants were able to select one or more of the following modes: biking outdoors, jogging or running, brisk walking, group fitness class (aerobic), group fitness class (muscle strengthening), swimming, hiking, and weight lifting (free weights or machines). If a participant engaged in an unlisted activity, they were able to select the “other” option and specify the activity using a text box. For each mode selected, participants were asked to indicate how many minutes they spent engaging in each exercise. Estimates of exercise volume (MET-minutes) were calculated by multiplying self-reported duration by metabolic equivalent of task (MET) values provided in the Compendium of Physical Activities (Ainsworth et al., 2011). These items were included in each electronic survey.

##### Demographics

In the baseline survey, participants reported age, level of education, gender, race, and ethnicity.

#### Statistical Analyses

Using SPSS, we isolated each participants' time series data to describe item characteristics (mean, standard deviation, and range) and repeated the procedures described for the R-technique analysis: (1) preliminary factor analyses (principle axis method with promax rotation applied to raw scores; factors retained based on eigenvalues ≥1.0) were conducted to determine initial sampling adequacy; (2) parallel analyses were conducted to statistically determine the number of factors to retain; and (3) final factor analyses were conducted with the number of factors constrained based on each individuals' parallel analysis. The use of parallel analysis has been previously determined to be an acceptable approach for P-technique factor analysis, as serial dependency of data does not negatively impact performance of the test (Lo et al., 2017). In three cases (participants 021, 022, and 029), the number of factors designated by the parallel analysis could not be extracted using participant's data. As we considered this work exploratory in nature, we chose to conduct the final factor analyses with one fewer factor than originally identified. In two cases (participants 024 and 015), a single item in each participant's data demonstrated zero variance (“achy” or “fit”). These variables were removed, respectively, from each participants' dataset prior to statistical analyses. Extracted communality plots were created to graphically visualize the person-specific structures (compared to the reference structure) in terms of how well each variable in the dataset is explained by the retained factors.

Personalized factor scores were estimated at each measurement time point by summing raw scores corresponding to item loading pattern and direction (i.e., negatively loaded items were subtracted from the total score) (DiStefano et al., 2009). In cases where data were missing, the participant's most recent estimated factor score was carried over. We then examined the intraindividual consistency of “factor 1” scores using a two-way random-effects model to compute the intraclass correlation coefficient (ICC) for absolute agreement. A higher ICC value (range 0.0–1.0) is indicative of greater consistency. In order to interpret the degree of consistency, values are classified as follows: <0.5 (poor), 0.5–0.75 (moderate), 0.75–0.9 (good), and >0.9 (excellent). The interpretation of these values was also considered in the context of 95% confidence intervals (Koo and Li, 2016).

## Results

### Study 1—R-Technique Factor Analysis

Participants (*N* = 572), on average (mean ± standard deviation), were comprised primarily of young adults (22 ± 6 years of age, 43.8% women) who reported exercising 4 ± 1 days per week. The racial make-up of the study sample reflected that of the university in general, such that 80.7% self-identified as non-Hispanic white (3.9% Asian, 8.8% African-American/Black, 3.5% Hispanic/Latino, 0.9% Native American, 0.9% Native Hawaiian/Pacific Islander and 1.2% indicating “other”). Although the student recreation center is open to any individual with a membership, the majority (81%) of respondents identified as undergraduate students.

The results of Study 1 provided the reference factor structure to which individual factor structures were compared. Participants' self-reported pre-exercise ratings for the 12 items were determined to be suitable for conducting factor analysis, as the KMO = 0.796 and Bartlett's test was statistically significant (chi square = 2720.256 (df 66), *p* < 0.001). The parallel analysis indicated four distinct factors (Table 1).

**Table 1:** Results of the parallel analysis to determine factor retention for R-technique exploratory factor analysis (EFA; Study 1).

**Initial factors**	**Initial eigenvalues from EFA**	**Parallel analysis raw data eigenvalues**	**Parallel analysis mean eigenvalue**	**Parallel analysis 95th percentile eigenvalue**
1*	3.843	3.341	0.264	0.326
2*	2.439	1.903	0.200	0.246
3*	1.385	0.851	0.150	0.188
4*	1.063	0.537	0.106	0.143
5	0.648	0.024	0.067	0.101
6	0.496	−0.073	0.029	0.057

*Asterisks denote factors that are statistically significant (raw eigenvalues > computed mean and 95th percentile eigenvalues)*.

The four factors explained nearly 60% of the rotated variance in the dataset and were identified as representing “health and fitness,” “fatigue,” “vitality,” and ‘physical discomfort” (Table 2). All items demonstrated sufficient loading magnitudes (0.585–0.891) with low cross-loading onto other factors (maximum absolute cross-loading = 0.106). These observations indicate that factors were distinct from one another, which allowed relative ease regarding interpretation.

**Table 2:** Descriptive statistics and R-technique exploratory factor analysis—pattern matrix (Study 1; *N* = 572).

**Item**	**Initial communalities**	**Mean ± SD (min-max)**	**Factor 1**	**Factor 2**	**Factor 3**	**Factor 4**
Strong	0.522	4 ± 1 (0-6)	**0.857**	−0.056	−0.057	0.080
Fit	0.474	4 ± 1 (0–6)	**0.751**	0.072	0.049	−0.019
Healthy	0.469	4 ± 1 (1–6)	**0.736**	−0.001	0.045	−0.076
Worn out	0.531	2 ± 1 (0–6)	−0.009	**0.849**	0.106	0.010
Drained	0.579	2 ± 1 (0–6)	−0.019	**0.808**	−0.063	0.016
Exhausted	0.469	1 ± 1 (0–6)	0.039	**0.724**	−0.072	−0.013
Lively	0.545	4 ± 1 (0–6)	−0.015	0.047	**0.891**	0.000
Energetic	0.535	4 ± 1 (1–6)	0.048	−0.063	**0.747**	0.028
Happy	0.356	4 ± 1 (0–6)	0.008	−0.006	**0.630**	−0.038
Achy	0.465	2 ± 1 (0–6)	0.020	0.026	0.028	**0.832**
Pain	0.383	1 ± 1 (0–6)	−0.050	−0.023	0.052	**0.708**
Stiff	0.310	2 ± 1 (0–6)	0.027	0.011	−0.102	**0.585**
Initial values		% Variance Eigenvalue	32.027	20.325	11.545	8.855
Rotated values		% Variance Eigenvalue	28.900	17.052	8.207	5.596

*Bold values meant to visually highlight which items strongly and uniquely loaded onto each factor.*

### Study 2—P-Technique Factor Analyses

Participants (*N* = 29) consisted primarily of young adults (24 ± 6y, BMI = 25.3 ± 3.3 kg/m^2^, 76% non-Hispanic White, 55% women). On average, participants self-reported engaging in 1240 ± 662 MET-Minutes per week of exercise.

We first assessed the degree of within-person variance in item ratings over time to determine if items described stable traits (i.e., minimal variance) or varying states. In many cases, participants responded using much of the 0–6 range. On average, the minimum rating across all items was 0.53 ± 0.96 and the maximum rating across all items was 4.94 ± 1.26. This indicates that item ratings demonstrated observable variance in affective and perceptual states across the 2-week monitoring period. However, the degree of discrimination between items differed across individuals. For example, participant 007 demonstrated the smallest difference between their highest item mean (“happy” = 3.36) and their lowest item mean (“pain” = 1.96), suggesting a higher degree of uniformity in ratings across all 12 items. In contrast, participant 028 demonstrated the largest difference between their highest (“healthy” = 5.93) and lowest (“pain” = 0.23) item mean. Of note, in 25 of the 29 individuals, an item representing physical discomfort (“achy,” “stiff,” “pain”) was observed to be the lowest item mean, of which “pain” was the lowest in 15 of these cases. Similarly, the highest item mean across individuals was often “happy” (16 cases); however, in four cases, an unfavorable item (e.g., “drained” or “exhausted”) demonstrated the highest mean among the 12 items.

The degree of discrimination within items also differed across individuals. There were only two cases where no temporal variance was noted for an item, in that two participants provided the same score for “achy” or “fit” at every measurement point over time. Excluding these cases, participant 006 demonstrated the most variance, such that average standard deviation across items was 2.24 (range: 1.61 for “fit” and 2.66 for “lively”). Conversely, participant 011 demonstrated an average standard deviation of 0.60 across all 12 items (range: 0.33 for “drained” and 0.82 for “lively”).

Sampling was determined to be adequate for all 29 individual factor analyses based on values pertaining to KMO (0.757 ± 0.062, min = 0.626, max = 0.892) and Bartlett's chi square (366.546 ± 112.038, min = 202.82, max = 679.65; all *p*'s < 0.001). When examining separate parallel analyses fit to the individual multivariate time series data for each participant, a median of three retained factors was observed among participants. Across all items, the average factor loading was 0.733 ± 0.150 (min = 0.406, max = 1.09). On average, the percentage of variance explained by each individual's first factor was 38.01 ± 9.50% (min = 21.96%, max = 66.92%).

**Figure 1:**
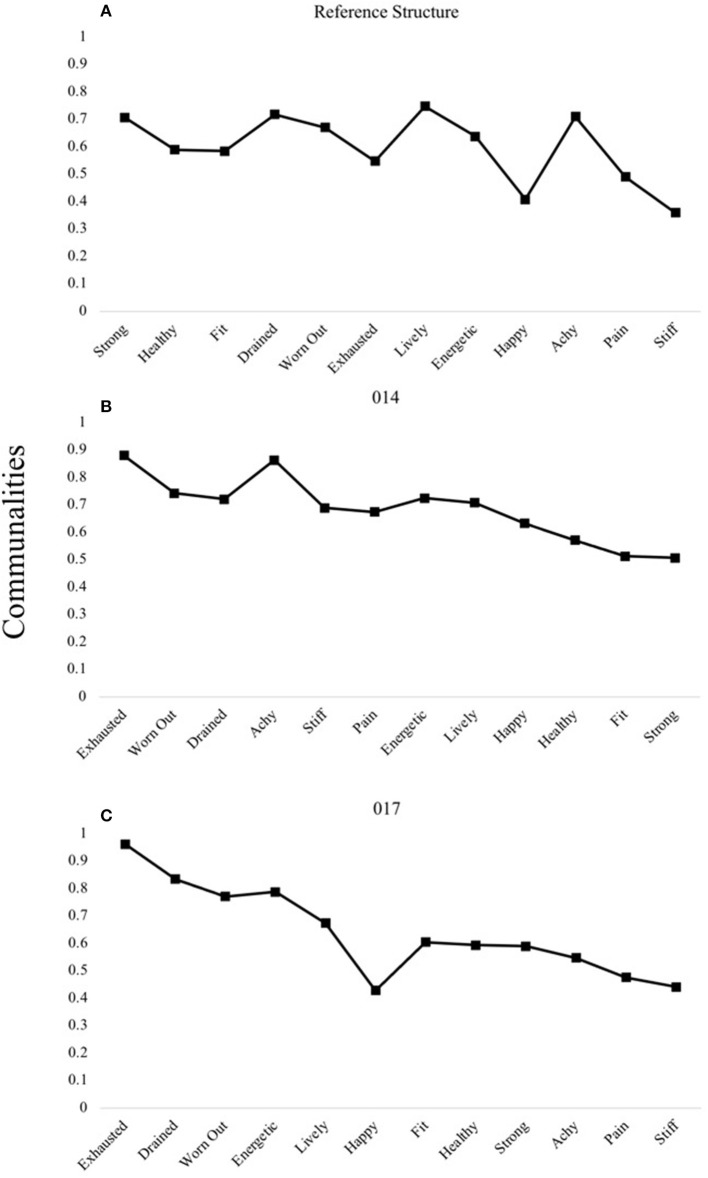
R- and P-technique four-factor models demonstrating unique patterns. The reference structure derived from R-technique factor analysis in Study 1 **(A)** is compared to the four-factor structure of participants 014 **(B)** and 017 **(C)** uncovered using P-technique factor analyses in Study 2. Communality scores ranging from 0 to 1 represent the degree to which each variable is explained by the resultant factors.

Only two participants' data were determined to have four factors, in line with the reference structure, according to the parallel analyses (Figure 1). In contrast to the reference structure, wherein the first factor was comprised of items pertaining to the “health and fitness” domain, the first factors in both person-specific structures were comprised of items relating to “fatigue.”

**Figure 2:**
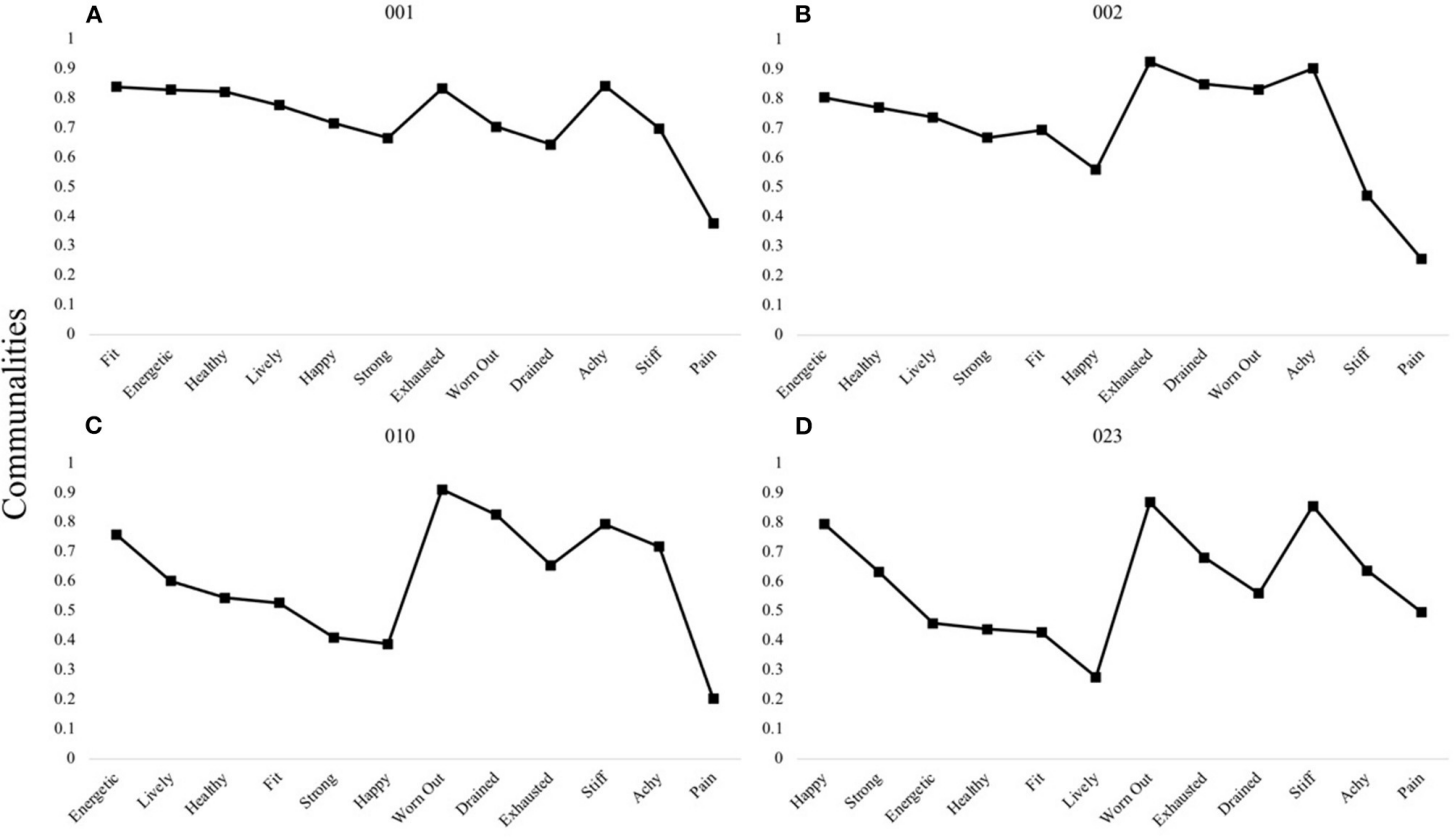
P-technique three-factor models demonstrating a single shared pattern. Participants 001 **(A)**, 002 **(B)**, 010 **(C)**, and 023 **(D)** are shown to demonstrate the most common three-factor structure observed among individuals: VH-F-D. V, “Vitality” domain comprised of items happy, energetic, lively; H, “Health and Fitness” domain comprised of items health, fit, strong; F, “Fatigue” domain comprised of items exhausted, worn out, drained; D, “Discomfort” domain comprised of items achy, stiff, pain. Communality scores ranging from 0 to 1 represent the degree to which each variable is explained by the resultant factors.

A three-factor structure was determined for a larger subset of participants (*n* = 11). Through interpreting the structures among a larger group of individuals, shared structural patterns in item loadings emerged under which three to four participants could be grouped. One pattern was characterized by all positively-valenced items (e.g., those representing “vitality” and “health and fitness” domains) loading onto the first factor, followed by separate factors representing “fatigue” and then “physical discomfort” (Figure 2).

**Figure 3:**
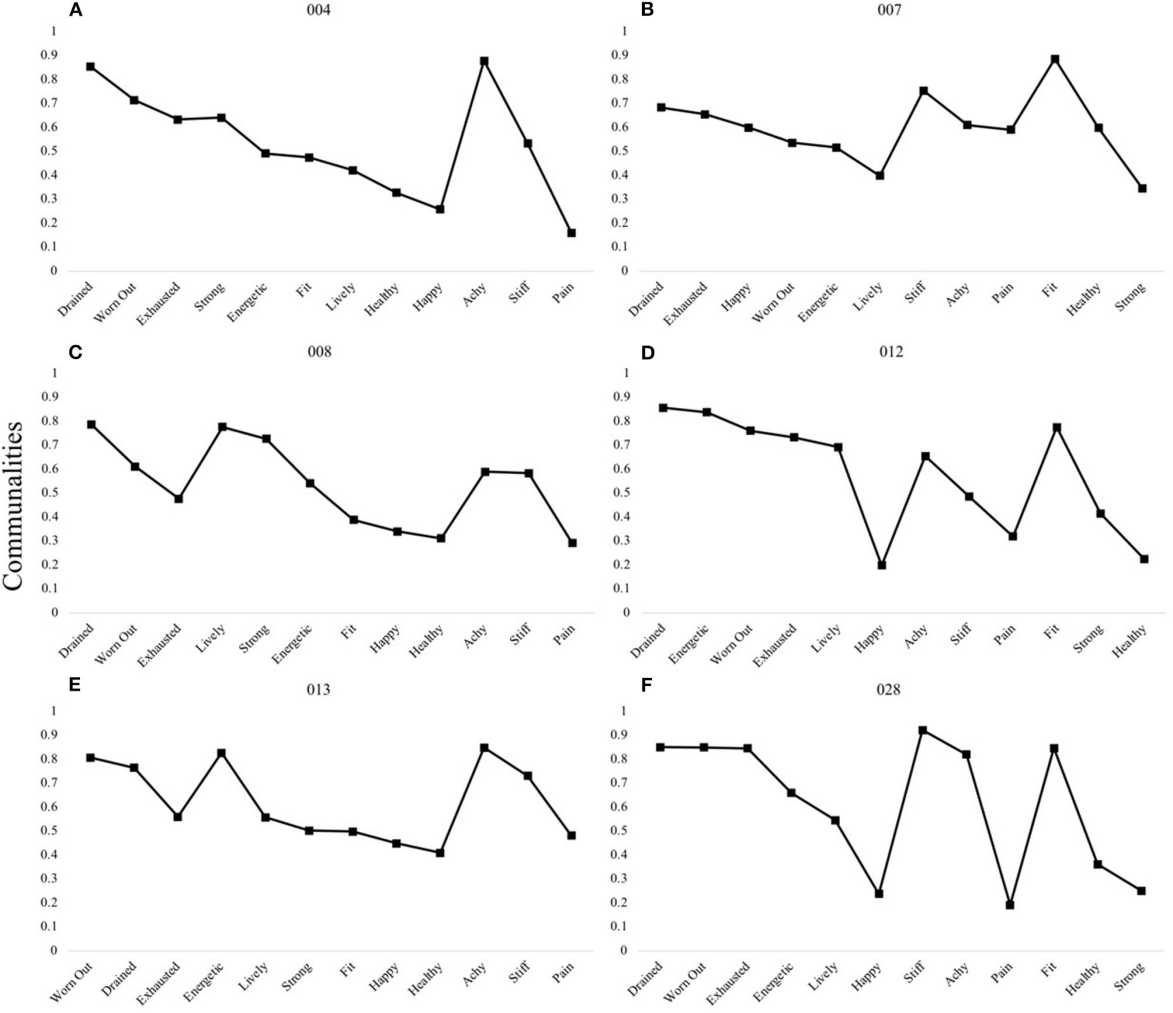
P-technique three-factor models demonstrating two shared patterns. Participants 004 **(A)**, 008 **(C)**, and 013 **(E)** are shown to demonstrate the shared F-VH-D pattern, whereas participants 007 **(B)**, 012 **(D)**, and 028 **(F)** demonstrate the VF-D-H pattern. V, “Vitality” domain comprised of items happy, energetic, lively; H, “Health and Fitness” domain comprised of items health, fit, strong; F, “Fatigue” domain comprised of items exhausted, worn out, drained; D, “Discomfort” domain comprised of items achy, stiff, pain. Communality scores ranging from 0 to 1 represent the degree to which each variable is explained by the resultant factors.

A second three-factor pattern was characterized by fatigue-specific first factors (Figures 3A,C,E). In the third pattern, items relating to “vitality” and “fatigue” domains loaded onto the first factor, with the second and third factors pertaining to “physical discomfort” and “health and fitness,” respectively (Figures 3B,D,F).

**Figure 4:**
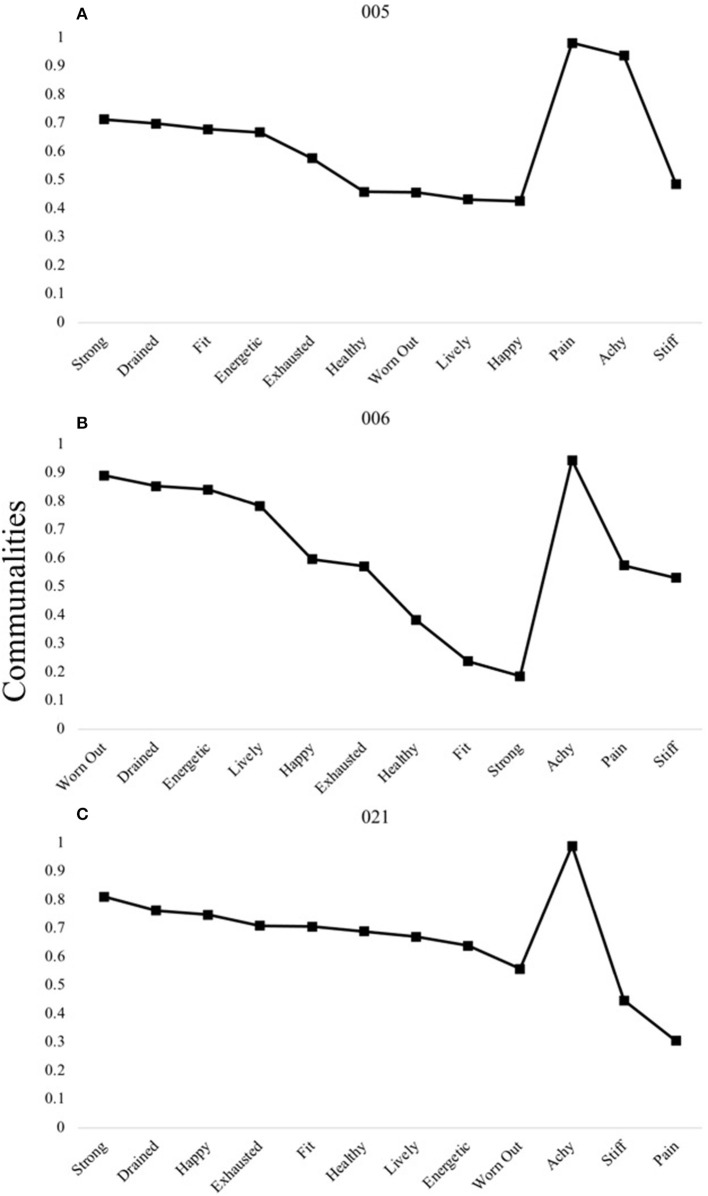
P-technique two-factor models demonstrating a single shared pattern. Participants 005 **(A)**, 006 **(B)**, and 021**(C)** are shown to demonstrate the most common two-factor structure observed among individuals: VHF-D. V, “Vitality” domain comprised of items happy, energetic, lively; H, “Health and Fitness” domain comprised of items health, fit, strong; F, “Fatigue” domain comprised of items exhausted, worn out, drained; D, “Discomfort” domain comprised of items achy, stiff, pain. Communality scores ranging from 0 to 1 represent the degree to which each variable is explained by the resultant factors.

A two-factor structure was determined for the majority of participants (*n* = 14), with shared patterns under which two to three participants could be grouped. Figure 4 shows the pattern shared by three individuals, which is characterized by all items loading onto the first factor except those pertaining to the “physical discomfort” domain.

**Figure 5:**
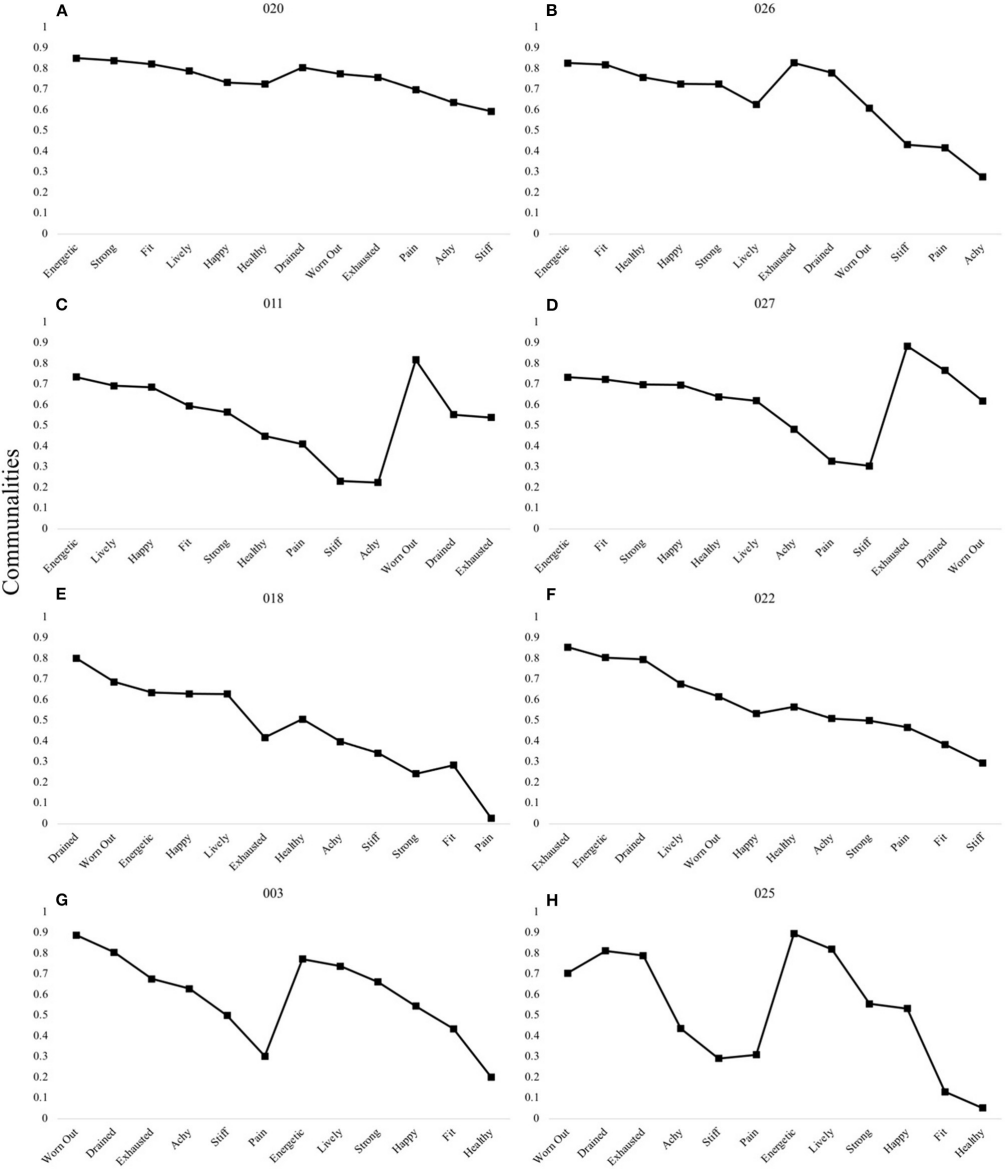
P-technique two-factor models demonstrating four shared patterns. The VH-FD pattern was observed in participants 020 **(A)** and 026 **(B)**. The VHD-F pattern was observed in participants 011 **(C)** and 027 **(D)**. The VF-DH pattern was observed in participants 018 **(E)** and 022 **(F)**. The FD-VH pattern was observed in participants 003 **(G)** and 025 **(H)**. V, “Vitality” domain comprised of items happy, energetic, lively; H, “Health and Fitness” domain comprised of items health, fit, strong; F, “Fatigue” domain comprised of items exhausted, worn out, drained; D, “Discomfort” domain comprised of items achy, stiff, pain. Communality scores ranging from 0 to 1 represent the degree to which each variable is explained by the resultant factors.

Figure 5 shows the remaining shared two-factor patterns. In these patterns, the first factors are comprised primarily of items pertaining to (1) all positively-valenced items (Panels A,B), (2) all domains except “fatigue” (Panels C,D), (3) to “fatigue” and “vitality” (Panels E,F), and (4) to all negatively-valenced items (Panels G,H).

**Figure 6:**
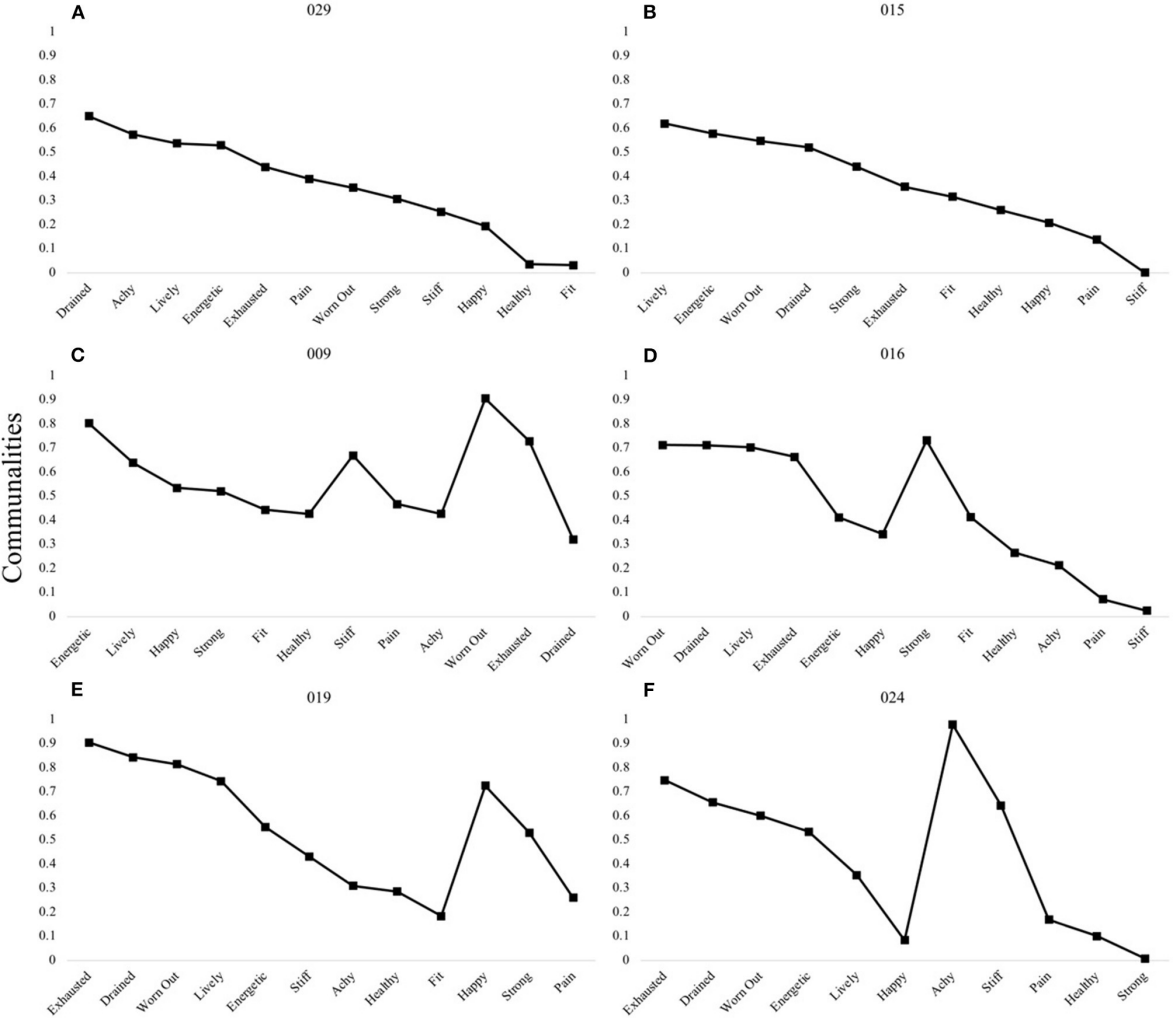
P-technique single factor models and unique multi-factor patterns. Participants 029 **(A)** and 015 **(B)** demonstrated a one-factor model. Four individuals presented with unique three-factor **(C)** or two-factor models **(D–F)**. Communality scores ranging from 0 to 1 represent the degree to which each variable is explained by the resultant factors.

Single factor structures are demonstrated in Figure 6 (Panels A and B). Additionally, this figure highlights the four participants who, based on their resultant factor structure, could not be grouped under shared patterns. This includes individuals with three-factor structures (Panel C) and two-factor structures (Panels D,E).

**Figure 7:**
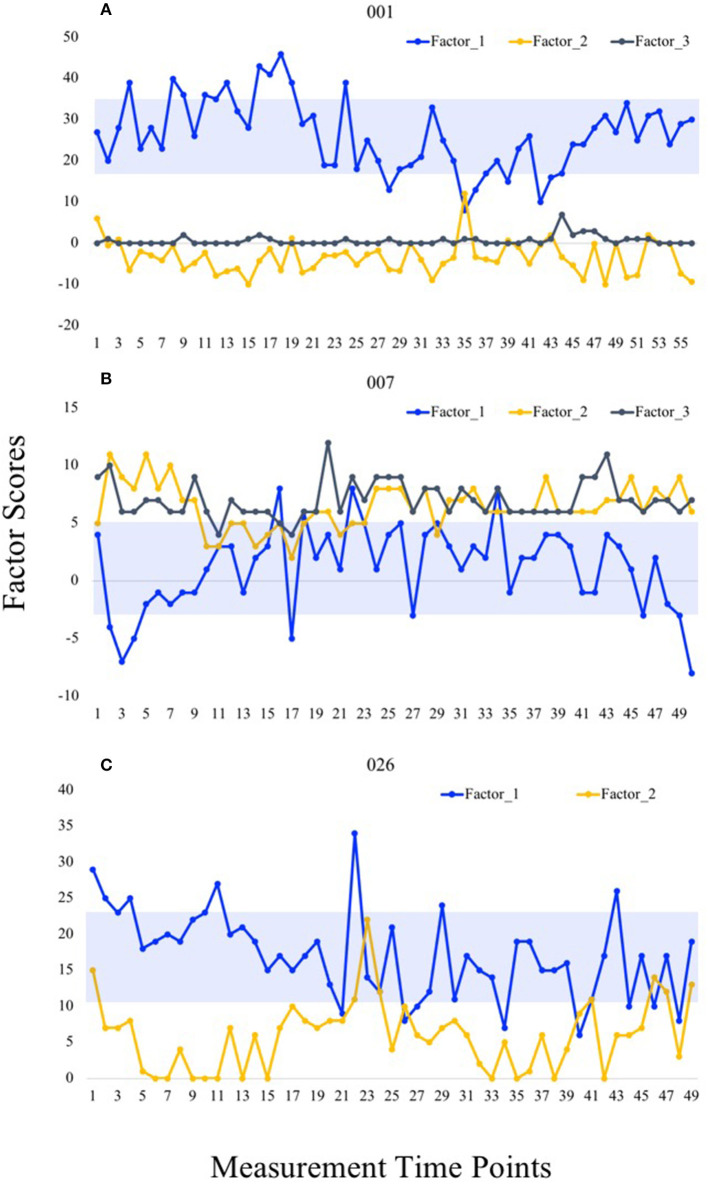
Temporal fluctuation in factor scores in representative cases. Factor scores calculated at each measurement time point are shown for Participants 001 **(A)**, 007 **(B)**, and 026 **(C)**. Blue boxes demonstrate one standard deviation from the mean in both directions.

The distribution of estimated “factor one” scores within each individual are demonstrated in Table 3. For 16 individuals, higher scores could be interpreted as representing a more favorable state (e.g., positive loadings of items pertaining to health/fitness and vitality with negative loadings of items pertaining to fatigue or physical discomfort). For the remaining 13 individuals, higher scores could be interpreted as less favorable (e.g., positive loadings for fatigue and physical discomfort). The ICC = 0.62, *p* < 0.001 (95% CI = 0.50–0.75), suggesting a moderate degree of consistency across estimated factor one scores. Figure 7 presents three representative cases to demonstrate fluctuation in calculated factor scores over the two-week measurement period. In all cases, higher first factor scores are indicative of a more favorable state. In Participant 001, higher second and third factor scores indicate more unfavorable states (i.e., more fatigue, discomfort; both domains are similarly accounted for together in the second factor of Participant 026). In Participant 007, higher second factor scores indicate a more unfavorable state (i.e., discomfort), whereas higher third factor scores indicate a more favorable state (i.e., greater perceptions of health and fitness).

**Table 3:** Description of within-person data distribution for estimated factor one scores for all participants (*N* = 29; Study 2).

**Participant ID (factor score range)**	**Mean ± SD (min-max)**	**Participant ID (factor score range)**	**Mean ± SD (min-max)**	**Participant ID (factor score range)**	**Mean ± SD (min-max)**
001 (0, 36)^a^	20 ± 7 (4, 36)	011 (−6, 36)^a^	22 ± 4 (15, 32)	021 (−18, 36)^a^	16 ± 9 (−5, 6)
002 (0, 36)^a^	16 ± 6 (3, 16)	012 (−18, 24)^a^	8 ± 7 (−5, 21)	022 (−18, 18)^b^	3 ± 9 (−17, 17)
003 (−6, 36)^b^	12 ± 11 (−6, 31)	013 (−6, 18)^b^	7 ± 4 (−1, 14)	023 (0, 30)^a^	11 ± 6 (0, 25)
004 (0, 18)^b^	8 ± 4 (1, 17)	014 (0, 18)^b^	11 ± 4 (3, 18)	024 (−12, 18)^b^	0 ± 4 (−8, 10)
005 (−18, 36)^a^	20 ± 8 (1, 33)	015 (−18, 36)^a^	19 ± 7 (3, 29)	025 (0, 36)^b^	14 ± 5 (3, 22)
006 (−18, 36)^a^	4 ± 16 (−18, 36)	016 (−18, 24)^b^	−3 ± 8 (−15, 12)	026 (0, 36)^a^	17 ± 6 (6, 22)
007 (−18, 18)^a^	1 ± 4 (−8, 8)	017 (0, 18)^b^	4 ± 3 (0, 12)	027 (0, 48)^a^	13 ± 6 (2, 15)
008 (−6, 18)^b^	5 ± 3 (−2, 15)	018 (−18, 24)^a^	10 ± 10 (−14, 24)	028 (−18, 18)^b^	−8 ± 6 (−17, 11)
009 (0, 36)^a^	20 ± 6 (6, 30)	019 (−12, 18)^b^	9 ± 5 (−6, 16)	029 (−24, 36)^b^	−16 ± 6 (−23, 14)
010 (0, 36)^a^	25 ± 4 (17, 31)	020 (0, 36)^a^	15 ± 9 (0, 36)		

a *Higher factor one score interpreted as more favorable state based on item loadings*.

b *Higher factor one score interpreted as a less favorable state based on item loadings*.

## Discussion

We conducted this research with the primary aim of comparing the nomothetical and idiographic approaches to modeling the relatively novel concept of readiness-to-exercise. Study 1 (nomothetic) therefore analyzed a cross-sectional data from 572 participants, suggesting a four-factor structure that explained 60% of the variance in readiness-to-exercise: “health and fitness;” “fatigue;” “vitality” and “physical discomfort.” Study 2 (idiographic) included a sample of 29 participants who completed the readiness-to-exercise scale multiple times, between 42 and 56 times: permitting intra-individual analyses. Our analyses suggested that many individuals displayed personal signatures, or profiles' of readiness-to-exercise that differed in structure from the nomothetic form: only two participants' personal signatures contained four structures as modeled in Study 1, whereas the majority were observed to have either two or three factors that were considered statistically significant in accordance with the parallel analyses. These findings raise important questions about how experiential data should be collected and modeled, for use in research (conceptual development and measurement) and applied practice (prescribing, monitoring)—as well as in more applied research (implementation, effectiveness).

From a conceptual viewpoint, readiness-to-exercise encompasses a variety of independent and interrelated psychological, physiological, and behavioral factors. When appropriately measured and appraised, indices of readiness-to-exercise offer promise in guiding in-the-moment modifications to exercise goals in response to an individual's changing circumstances over time. The application of factor analyses serves to reduce the dimensionality of multivariate data to observe fewer latent variables and the degree to which they explain variance in the full dataset (Cattell, 1952). From a practical perspective, Cattell (1952) noted that, in order to test a given hypothesis using standard experimental procedures, the researcher must first identify the variable (or variables) of most importance, often from a large array of potential variables. According to the R-technique factor analysis conducted in the first study, variables conceptualized to represent the “health and fitness” dimension (“strong,” “fit,” “healthy”) collectively explained the most (29%) rotated variance in the dataset. Based on Cattell's reasoning, we might assume that, as it is considered the most important factor, differentiation in this factor score (i.e., high vs. low) would better discriminate which individuals should experience a standardized exercise session more favorably or unfavorably compared to “discomfort” factor scores, which explained the least variance. Additionally, items loading on this latter factor (“pain,” “achy,” “stiff”) were scored lower compared to items that loaded on the other three factors. This finding is most likely explained by the young age and relatively high exercise level of the sample population, as physical inactivity is a predominant risk factor for chronic pain conditions (Landmark et al., 2011). Further, regular exercise has been demonstrated as an efficacious strategy for alleviating pain across various conditions (Biodonde et al., 2014; Fransen et al., 2015; Searle et al., 2015).

The R-technique structure observed in the current study contrasts with that demonstrated in the previous R-technique factor analysis of Strohacker and Zakrajsek (2016), wherein the analogous factor, comprised of just two items (“healthy” and “fit”), explained the least amount of rotated variance (2%) in their four-factor model. One likely explanation for these contrasting findings, given the similar student sample populations in both studies, is that variables relating to mood, emotional, and energetic arousal states were overrepresented in the earlier study. Through their systematic development of an item pool, Strohacker and Zakrajsek (2016) combined readiness-related words frequently listed by participants with items from commonly used psychometric instruments in exercise psychology research, which do not ascertain physiological states (e.g., sickness, hunger, and pain) or perceptions of fitness (e.g., endurance, strength, flexibility). Having fewer related items reduces the potential explanatory strength of a given factor. An alternative possibility is that differences in first factor item composition could be due to the context of data collection. For the current study, participants provided *integral* item ratings (i.e., in a pre-exercise context), as compared to the incidental ratings (i.e., in a classroom setting) provided by participants in the previously published work. Conceptually, readiness-to-exercise is not intended to be a predictor of future of exercise behavior, but rather, a predictor of an *impending* exercise experience (i.e., the decision to exercise is about to been enacted). In this regard, determining whether or not population-level structures hold across measurement contexts is particularly valuable. Contextual differences may also extend to the level of physical demand proposed. In their qualitative analysis of open-ended survey responses, Strohacker et al. (2019) noted that the theme pertaining to perceptions of fitness only emerged when respondents were asked to describe how they would need to feel to complete a 60-min jog, and not when asked to consider readiness to complete a 10-min slow stroll. The influence of context on resultant factor structures should be subsequently explored.

The results of the current study demonstrate that structural features of factors modeled using P-technique factor analysis display heterogeneity when compared to a reference structure achieved through the traditional R-Technique. This finding first suggests that the most important subjective variables (i.e., those that explain the most variance in the data) differ between individuals. Second, by modeling a set of factors using time-series data, researchers can uncover each individual's *dynamic structure* to reveal patterns of covariation that yields unique insight into a person's relevant preconditions, behaviors and resultant experiences (Wright, 2017). Our study provides proof-of-concept for the existence of numerous, distinctive patterns in structural features, occurring at least in regards to individuals' subjective precondition. In viewing the variability demonstrated in the communality plots, there is little doubt that “interpersonal signature” is a fitting term to apply. When mapping all factors scores over time, additional, person-specific information emerges. For example, in viewing the first factor scores (both calculated by summing all six positively-valenced items) in Participants 001 and 026, we note that the former generally presented with higher scores (26 ± 9) compared to the latter (17 ± 6). Additionally, we also note that Participant 007 generally presented with higher scores for discomfort (second factor) than Participant 001 (third factor). Person-specific differences in central tendency and spread of factor scores should be accounted for, as it has previously been demonstrated that modeling trait-level patterns of instability as a construct improves physical activity prediction (Dunton, 2017).

The observation that multiple individuals could be represented by a particular structural pattern also aligns with the findings of Wright et al. (2016), who were able to discuss their results (which did not pertain to exercise or physical activity, however), using five “exemplar” cases to represent structures from 25 individuals. It is important, however, to consider these results from both studies in the context of the relatively small sample populations included (*N*'s < 30). It is unlikely that this work was sufficiently powered to demonstrate either the full array of truly unique structural configurations or the number of representative configurations, under which numerous person-specific structures reasonably cluster. Further, as the sample was relatively homogenous regarding age, activity level, and race, we did not analyze the data to understand if those demonstrating similar interpersonal signatures also shared key demographic or habitual behavior features. To answer these important questions, future work likely requires larger and more diverse sample populations. Nevertheless, given the theoretical possibility that individuals may not all experience the world in the same way—varying for example in interoceptive sensitivity and cultural reference-points—then our findings reinforce the argument that measuring and studying such experiences using nomothetic assumptions may be inappropriate for both researchers and practitioners alike. Findings may be unreliable, or invalid, in relation to the underlying reality, but participants may also feel alienated and poorly represented by the questions and resulting “insights.” While, in the case of the current study, we are referring to subjective feelings and physical cues, researchers have previously surmised that individuals ascribe meanings to situations that can either be broadly shared by others or that are particularly idiosyncratic—the combination of which reveals person-specific dispositions and situation-behavior signatures (Fournier et al., 2008).

The demonstration of within-person variability, not only in singular item ratings, but also in estimated factor one scores further highlights that individuals in our study experienced and reported changing circumstances. Further, the ICC regarding estimated factor one scores within individuals fell below the threshold for “good” consistency (ICC ≥ 0.75), suggesting that individuals experience a degree of variance even over a relatively short time period. In other words, the factor that explains the most variance for each individuals' data better represents dynamic states, rather than static traits. This finding lends support for refining FNLP to account for individuals' changing circumstances. In particular, we propose that applying the P-technique should allow researchers to objectively identify individualized models of readiness-to-exercise in order to determine the smallest number of the most important variables to monitor over time, for each person. This proposed approach is in contrast to the varied and relatively unstructured approaches to operationalize readiness in the existing FNLP-based research. For example, in reviewing time-series data for Participant 001, time-point 35 likely represents a vulnerable period, wherein scores for positively-valenced items (i.e., energetic, happy, strong, and fit) are lower than normal and scores for fatigue-related items are higher than normal. It is possible that factor scores at time-points 3 and 23 for Participants 007 and 026, respectively, may similarly signal an increased vulnerability to negative acute responses to exercise. While such speculations need to be validated through experimental or observational data, the approach provided in the current study can serve as a feasible starting point for developing research designs to test such hypotheses.

Overall, the success of person-adaptive approaches for exercise programming hinges on participant “buy-in” to put forth sufficient efforts for data collection. That is, individuals must be willing to: (1) diligently self-monitor behavioral outcomes and/or routinely wear and care for physical activity trackers; (2) provide prompt and unbiased psychological and perceptual feedback consistently over time; and (3) be sufficiently responsive to inquiries and new directions. Ultimately, participants need to put forth mental effort above and beyond that of daily living, in essence, to allow a clinician or researcher to effectively guide dynamically personalized decisions in the best interest of participants' in-the-moment circumstances. Therefore, the experience of generating data (as well as the exercise itself) needs to feel relevant, worthwhile and rewarding. Thus, in appreciation of this potential burden, researchers must strive to use collected data appropriately to inform decisions based on individuals' most salient and informative constructs. Based on the results of the current study—and as argued in the supporting methodology papers that we used to design this study—P-technique factor analysis of data collected *via* ecological momentary assessment offers a powerful process that can be used in early-stage development to reduce multivariate data and view potentially meaningful factors through an idiographic lens.

In discussing the findings of the current study, it is important to consider a concern, raised by Borkenau and Ostendorf (1998), that heterogeneity derived from P-technique analyses that departs from a reference structure may simply be an artifact of having fewer measurement points to analyze. The same authors nonetheless recognized that it would be exceptionally difficult to obtain sufficient time series data in a single person for robust comparisons to be made between R- and P-techniques. Alternatively, we propose that a more efficient approach for researchers is to direct efforts toward substantiating the utility of person-specific structures of readiness derived from P-technique factor analysis. For example, empirical modeling procedures could be applied to determine relationships between first factor scores and relevant outcomes (e.g., exercise-related effort, exertion, affect, and performance appraisals). As an example of this approach, using point-biserial correlations, Wright et al. (2016) observed that estimated first factor scores were associated with key behavioral outcomes in the target sample population. The informational utility of uncovered factor structures could also be explored through mixed methods designs. For example, individuals' interpersonal readiness signatures could be objectively constructed using P-technique factor analysis and presented to individuals in one-on-one interviews to gauge their perceptions regarding saliency as well as likely impact on exercise-related behavior and experiential outcomes. Systematic research efforts toward defining and testing key intervention components—in our case person-specific models of readiness-to-exercise—*via* experimental and qualitative designs are strongly promoted by experts in health psychology to more efficiently develop and refine promising, evidence-based behavioral treatments prior to examining efficacy in randomized controlled trials (Czajkowski et al., 2015). We also note the possibility of researching if-and-when participants' “personal-signature” factor structures may change, for example in response to learning or training: which seems both a plausible consequence of these findings and indeed a possibility suggested by researchers assessing interventions to develop interoception (Çöl et al., 2016; Navarro-Haro et al., 2019) and mindfulness (Farb et al., 2013; Haase et al., 2016).

The current research does present with several limitations. First, we do not propose that the original item pool for both studies represents all potential constructs underlying readiness-to-exercise, as these data had already been collected and, thus targeted for secondary analyses. The results should be interpreted more as proof-of-concept that idiographic factor structures can depart from those derived from nomothetic approaches: thus, requiring further research using purposefully collected and more recent data. Specifically, such data (or at least a portion thereof) should be integral (i.e., collected specifically within the pre-exercise context, as was accomplished in the first study), because the data analyzed in the second study better represent incidental measures of readiness-related constructs. Second, because the sample populations of both studies mainly consist of university students, it cannot be assumed that the uncovered structural features are representative of other populations who may perceive readiness-to-exercise differently, such as older adults, those diagnosed with chronic disease, or adults who must manage additional stress-producing life priorities (e.g., full-time employment, child or elder care). Further, as the participants primarily identified as non-Hispanic white, these findings cannot necessarily be extrapolated to individuals that identify with other (often minoritized) racial categories and ethnicities.

In conclusion, the current findings demonstrate that interpersonal signatures (and clusters of similarly-structured signatures among individuals) of readiness-to-exercise generated *via* P-technique factor analyses often depart from a generalized structure of readiness-to-exercise (i.e., differing in factor number, percentage of variance explained per factor, and item loadings within each factor). Uncovering dynamic state differences within an individual over time opens opportunities to more precisely identify important components to measure, which may be shared or unique across individuals, and used to guide personally-adaptive exercise programming. P-technique factor analysis offers a preliminary means of modeling idiographic structures and features of multivariate data that can be collected with relative ease using smartphone technology. Applying this process may help exercising individuals and practitioners begin to answer complex questions (i.e., which precondition—my energy level or my perception of physical discomfort—is more predictive of a subsequent exercise experience) that may otherwise be difficult to articulate “off the cuff,” in absence of interpretable data. Such an approach would depart from current practices of utilizing a single, practitioner-chosen variable or relying on participants' personal (and potentially uninformed or uncritical) choices. Subsequent efforts to understand both the predictive and informational utility of individuals' uncovered factors aligns with recent calls by experts to progress toward person-specific interventions for both sport and general physical activity promotion.

## Data Availability Statement

The raw data supporting the conclusions of this article will be made available by the authors, without undue reservation.

## Ethics Statement

The studies involving human participants were reviewed and approved by The University of Tennessee, Knoxville Institutional Review Board. The patients/participants provided their written informed consent to participate in this study.

## Author Contributions

The collection of data utilized in Study 1 was designed and carried out by KS and RZ. The collection of data utilized in Study 2 was designed and carried out by KS. All authors contributed to data analysis and interpretation in the current study, as well as to all aspects of manuscript preparation.

## Conflict of Interest

The authors declare that the research was conducted in the absence of any commercial or financial relationships that could be construed as a potential conflict of interest.
